# Patterns of Spontaneous Brain Activity in Amyotrophic Lateral Sclerosis: A Resting-State fMRI Study

**DOI:** 10.1371/journal.pone.0045470

**Published:** 2012-09-20

**Authors:** ChunYan Luo, Qin Chen, Rui Huang, XuePing Chen, Ke Chen, XiaoQi Huang, HeHan Tang, QiYong Gong, Hui-Fang Shang

**Affiliations:** 1 Department of Neurology, West China Hospital, SiChuan University, Chengdu, Sichuan, China; 2 Huaxi MR Research Center, Department of Radiology, West China Hospital, SiChuan University, Chengdu, Sichuan, China; 3 Division of Medical Imaging, Faculty of Medicine, University of Liverpool, Liverpool, United Kingdom; The University of Melbourne, Australia

## Abstract

By detecting spontaneous low-frequency fluctuations (LFF) of blood oxygen level–dependent (BOLD) signals, resting-state functional magnetic resonance imaging (rfMRI) measurements are believed to reflect spontaneous cerebral neural activity. Previous fMRI studies were focused on the examination of motor-related areas and little is known about the functional changes in the extra-motor areas in amyotrophic lateral sclerosis (ALS) patients. The aim of this study is to investigate functional cerebral abnormalities in ALS patients on a whole brain scale. Twenty ALS patients and twenty age- and sex-matched healthy volunteers were enrolled. Voxel-based analysis was used to characterize the alteration of amplitude of low frequency fluctuation (ALFF). Compared with the controls, the ALS patients showed significantly decreased ALFF in the visual cortex, fusiform gyri and right postcentral gyrus; and significantly increased ALFF in the left medial frontal gyrus, and in right inferior frontal areas after grey matter (GM) correction. Taking GM volume as covariates, the ALFF results were approximately consistent with those without GM correction. In addition, ALFF value in left medial frontal gyrus was negatively correlated with the rate of disease progression and duration. Decreased functional activity observed in the present study indicates the underlying deficits of the sensory processing system in ALS. Increased functional activity points to a compensatory mechanism. Our findings suggest that ALS is a multisystem disease other than merely motor dysfunction and provide evidence that alterations of ALFF in the frontal areas may be a special marker of ALS.

## Introduction

Amyotrophic lateral sclerosis (ALS) is a devastating neurodegenerative disease characterized by progressive atrophy and weakness of the bulbar, limb, and respiratory muscles, with a median survival of 3–5 years from symptom onset. [1] Although ALS is predominantly a motor-system-degeneration disease, cognitive and behavioral symptoms have also been observed in ALS patients [2]. Almost half of ALS patients may have variable degrees of cognitive impairment with typical frontal executive deficits. Fourteen percent of ALS patients may have a clinical subtype of frontotemporal lobar degeneration called frontotemporal dementia [3]. Generalized sensory system abnormalities which were confirmed by a few large sample studies [4], [5] support the theory that ALS is a multisystem neurodegenerative disorder. Widespread cerebral degeneration of neurons in typical ALS cases [6]–[8] have been observed in post-mortem studies. Moreover, previous studies have suggested that dysfunction of extra-motor systems occurred in ALS patients, such as neurobehavioral dysfunction or frontotemporal dementia, might have negative effects on outcomes of patients [2], [9], [10]. Current treatments for ALS have limited effectiveness and are far from satisfactory. The lack of insight into the neuropathology of ALS is holding back improvements in ALS therapies [11]. Thus, it is of utmost importance to elucidate functional changes in both motor and extra-motor systems of the brain of ALS patients.

With the advantage of being noninvasive, functional magnetic resonance imaging (fMRI) has been extensively applied to study ALS. Most previous fMRI investigations on ALS focused on motor-related neural activity. In studies of motor execution, increased cortical activation has consistently been found in the sensorimotor cortex [12]–[17], premotor areas [12]–[18], and supplementary motor areas [12]–[17]. Some brain areas including the inferior parietal lobule [15], [17], [19], anterior cingulated cortex [14], [16], cerebellum [14], [17], basal ganglia [14], [20], and thalamus [18] were less consistent among various studies. The increased cortical activation is commonly thought to be a result of decreased intracortical inhibition, which may act in a compensatory role [12]. On the other side, a significant decrease of cortical activation caused by the presence of bulbar signs has also been observed when ALS patients performed vertical tongue movement [18], [21]. This finding indicates that deficits in compensatory processes in patients with bulbar involvement can be the reason of the faster time-course in this subgroup. However, the different patterns of performances or tasks make it difficult to interpret the biological mechanisms of ALS and hinder the application of across-population comparisons.

Unlike the traditional fMRI, the recently developed resting-state fMRI (rfMRI) techniques avoid potential performance confounds that may be present in subjects with cognitive or motor impairments since rfMRI does not require the subjects to perform any task [22], [23]. Spontaneous low-frequency fluctuation (LFF; <0.08 Hz) of the blood oxygen level dependent (BOLD) signals during rest has been found to be of physiological importance and related to intrinsic neuronal activity [24]–[26]. The measurement of rfMRI BOLD signals is a promising approach to assess regional and neural circuitry function at rest, which has been widely used to study various psychiatric and neurological disorders, such as schizoprehnia [27], [28], depression [29], Alzheimer's disease [30], Parkinson’s disease [31], [32] and ALS [23], [33]–[38]. Using the regions-of-interest (ROI) based approach, several groups reported altered functional connectivity in the motor areas of ALS patients [23], [33], [35], [36], as well as in prefrontal and thalamic regions [33]. However, among these studies, the prior selected seeds were mainly located in the motor network, and prior selection of the time series of particular subregions with which to correlate imposes anatomical restriction on the analysis of network connectivity. Little is known about the neural function beyond the motor network. Only few studies have found that the intrinsic activities altered within sensory-motor network [34], [37], default-mode network [37], [38] and frontoparietal network [38] in ALS patients. The patterns of intrinsic brain activity in ALS patients across the whole brain are still poorly understood.

Zang et al. [39] developed an index called amplitude of LFF (ALFF) to assess the amplitude of resting-state spontaneous brain activity, by calculating the square root of the power spectrum in a low-frequency range (usually 0.01–0.08 Hz). This approach eliminates the need to specify seed locations and can be applied to conduct the whole-brain voxel-wise analysis of cerebral function during the resting state. By measuring ALFF, several recent studies have successfully shown that baseline brain activities were altered in different physiological states of the brain [28], [30], [40]–[42]. To the best of our knowledge, there is no report on ALFF alterations in ALS.

In the present study, we investigated ALS-related alterations of brain activities during the resting state using ALFF, and its correlations with clinical features. We hypothesized that (1) brain activity would be altered in ALS, which is not restricted to the motor system and that (2) ALFF changes in these areas may be correlated with ALS clinical manifestations.

## Materials and Methods

### Participants

Twenty probable or definite sporadic ALS patients were recruited consecutively from the neurology department of West China Hospital of Sichuan University. ALS patients were diagnosed in accordance with the El Escorial revised criteria [43]. Sporadic ALS was defined as ALS without family history of ALS. All ALS patients were right-handed. Patients were excluded if they had other neurological diseases or psychiatric problems such as cerebrovascular disorders, hydrocephalus, intracranial mass, psychiatric hospitalization, substance abuse, or traumatic brain injury. All ALS patients underwent a thorough history and physical examination on the day of the study. The severity of the disease was assessed by the ALS functional rating scale (ALSFRS) questionnaire [44]. Muscle strength was scored using the Medical Research Council (MRC) scale from 0 to 5 [45]. Disease duration was calculated from symptom onset to scan date in months. Disease progression rate was obtained through dividing the ALSFRS scores by disease duration.

The control group was composed of sex and age-matched healthy right-handed individuals, whom were free of neurological or psychiatric diseases as assessed by a neurologist. None of them received psychotropic medication.

The present study was approved by the local Ethics Committee of West China Hospital of Sichuan University. Written informed consents were obtained from all subjects.

### MRI Acquisition

MRI was performed on a 3.0 Tesla (T) MR imaging System (Excite; GE, Milwaukee, WI) by using an eight-channel phased-array head coil. High resolution T1-weighted images were acquired via a volumetric three-dimensional spoiled gradient recall sequence (TR = 8.5 msec, echo time = 3.4 msec, flip angle = 12°, slice thickness = 1 mm). Field of view (240×240 mm^2^) was used with an acquisition matrix comprising 256 readings of 128 phase encoding steps that produced 156 contiguous coronal slices, with a slice thickness of 1.0 mm. The final matrix size of T1-weighted images was automatically interpolated in-plane to 512×512, which yielded an in-plane resolution of 0.47×0.47 mm^2^. MR images sensitized to changes in BOLD signal levels (TR = 2000 msec, echo time = 30 msec, flip angle = 90°) were obtained via a gradient-echo echo-planar imaging sequence (EPI). The slice thickness was 5 mm (no slice gap) with a matrix size of 64×64 and a field of view of 240×240 mm^2^, resulting in a voxel size of 3.75×3.75×5 mm^3^. Each brain volume comprised 30 axial slices and each functional run contained 200 image volumes. The fMRI scanning was performed in darkness, and the participants were explicitly instructed to relax, close their eyes and not fall asleep (confirmed by subjects immediately after the experiment) during the resting-state MR acquisition. Ear plugs were used to reduce scanner noise, and head motion was minimized by stabilizing the head with cushions.

### fMRI Data Analysis

Functional image preprocessing and statistical analysis was carried out using the SPM8 (Welcome Department of Imaging Neuroscience, London, UK; http://www.fil.ion.ucl.ac.uk). The first ten volumes of functional images were discarded for the signal equilibrium and participants’ adaptation to scanning noise. Then, the remaining EPI images were preprocessed using the following steps: slice timing, motion correction, spatial normalization to the standard Montreal Neurological Institute (MNI) EPI template in SPM8 and resample to 3×3×3 mm^3^, followed by spatial smoothing with 8-mm full-width at half-maximum (FWHM) Gaussian kernel. According to the record of head motions within each fMRI run, all participants had less than 2 mm maximum displacement in the x, y, or z plane and less than 1° of angular rotation about each axis.

ALFF was calculated using REST [46] (http://restfmri.net/forum/rest_v17) with a voxel-based approach similar to that used in earlier studies [28], [42]. After preprocessing, the time series for each voxel was filtered (band pass, 0.01–0.08 Hz) [26] to remove the effects of very-low-frequency drift and high frequency noise, e.g., respiratory and heart rhythms. Then, the filtered time series was transformed to a frequency domain using a fast Fourier transform (FFT) (parameters: taper percent = 0, FFT length = shortest) and the power spectrum was then obtained by square-rooted FFT and averaged across 0.01–0.08 Hz at each voxel. This averaged square root was taken as the ALFF. For standardization purposes, the ALFF of each voxel was divided by the global mean ALFF value to standardize data across subjects.

### VBM Analysis

Recent studies of fMRI have suggested that functional results could potentially be influenced by structural differences among groups [47]. To explore the possible effect, we performed a voxel-based morphometry (VBM) analysis for structural images. In this study, we used the diffeomorphic anatomic registration through an exponentiated lie algebra algorithm (DARTEL) [48] to improve the registration of the MRI images. DARTEL has been shown to be more sensitive than standard VBM methods [49]. Before segmentation, we checked for scanner artifacts and gross anatomical abnormalities for each subject; and the image origin was set to the anterior commissure. Then, MR images were segmented into gray matter (GM), white matter (WM) and cerebrospinal fluid using the unified segmentation model in SPM8 [50]. In a next step, a GM template was generated through an iteratively nonlinear registration (DARTEL; [48]). The GM template was normalized to MNI space and the resulting deformations were applied to the GM images of each participant. Finally, spatially normalized images were modulated to ensure that the overall amount of each tissue class was not altered by the spatial normalization procedure, and smoothed with an 8-mm FWHM Gaussian kernel.

### Statistical Analysis

Two-sample t-tests were performed to assess the differences in age, sex and handedness between ALS patients and controls, A two-tailed *p* value of <0.05 was deemed significant.

The differences of voxel-based ALFF between ALS patients and controls were analyzed using SPM8 with a two-sample t-test. Significant differences were set at the threshold of voxel-wise t>3.32 (P<0.001) and cluster level of p<0.05 corrected by family-wise error (FWE) correction. The MNI coordinates were transformed to Talairach coordinates using mni2tal. The results were presented using the voxel of peak significance.

Voxel-based comparisons of gray matter volume were performed between groups using two sample t-tests with total intracranial volume as covariates. The significance of group differences was set at the threshold of voxel-wise T>3.32 (p<0.001) and cluster level of p<0.05 corrected by FWE correction.

Furthermore, we reanalyzed the difference of ALFF between two groups by entering the GM probability maps as voxel-wise covariates [47] using the Biological Parametric Mapping toolbox [51] (http://fmri.wfubmc.edu/software/Bpm).

The average ALFF of all voxels in the abnormal areas revealed by voxel-based analysis was extracted separately using the volume of interest (VOI) in SPM8. Pearson correlation coefficients were calculated to evaluate the relationship between clinical variables (disease duration, disease progression rate and ALSFRS scores) and the mean ALFF within the VOIs, and the significance was set at P<0.05 (two-tailed).

## Results

Demographic and clinical data for ALS patients and controls were given in **Table 1**. There were no significant differences in age, gender and handedness between the two groups.

**Table 1:** Demographic and Clinical Characteristics of ALS Patients and Control Subjects.

	*ALS*	*Control*	*P-value*
N	20	20	−
Gender (male: female)	13∶07	14∶06	0.74
Mean age (years)	45.30±9.95	47.05±9.89	0.58
Handedness for writing (R:L)	20∶00	20∶00	–
Site of onset (bulbar: cervical: lumbar)	4∶10:06	–	–
Mean age of onset (years)	44.69±10.18	–	–
Mean disease duration (months)	15.20±10.37	–	–
Progression rate, mean a	3.83±2.64	–	–
ALSFRS^b^, mean	31.85±5.39	–	–

a Progression rate = (40-ALSFRS)/disease duration (months).

b ALSFRS-ALS functional rating scale.

**Figure 1:**
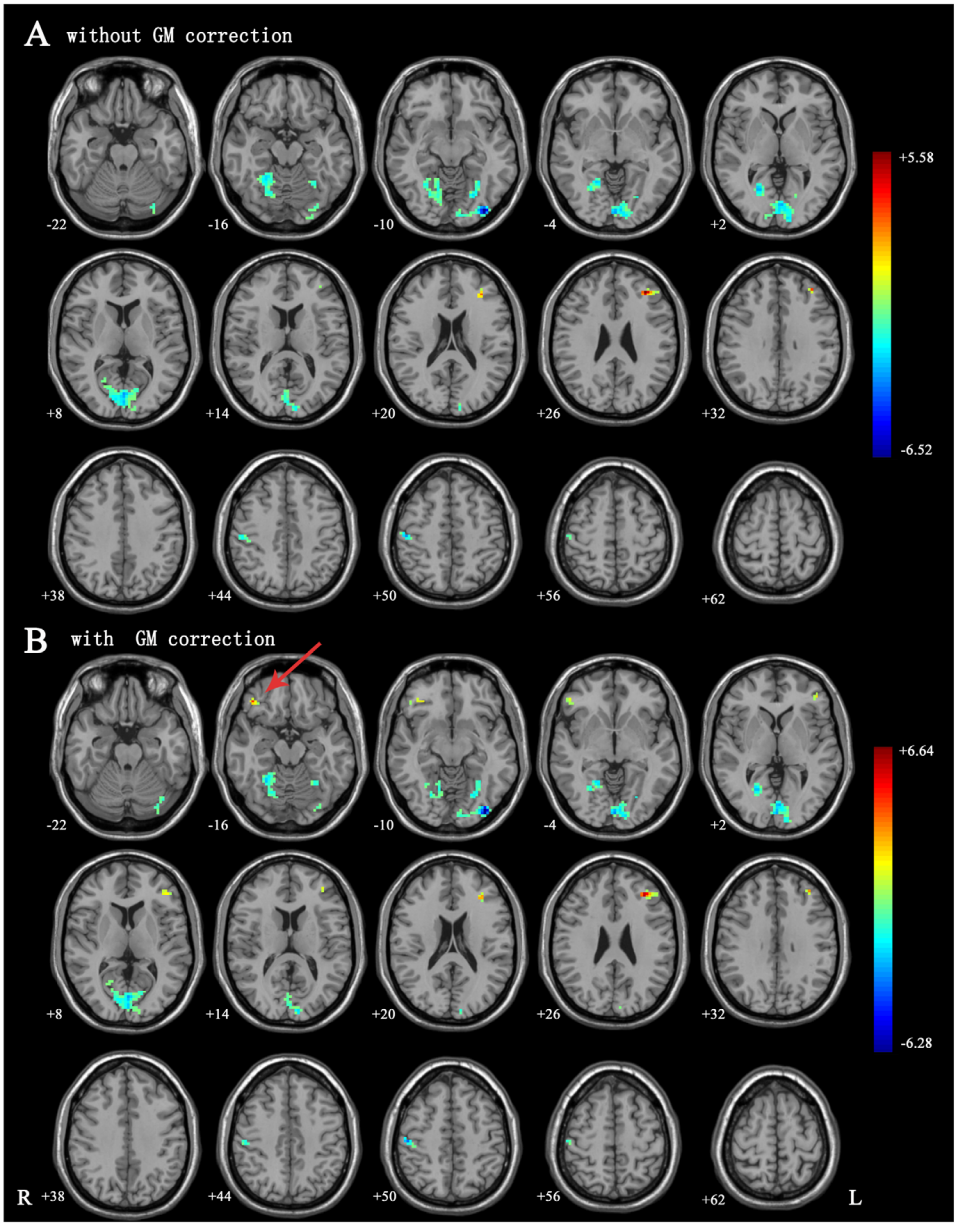
Differences in the spatial patterns of ALFF. (A) ALFF differences between ALS and control groups without GM correction. There were significant decreased ALFF in visual cortex, fusiform gyrus and right postcentral gyrus, as well as increased ALFF in left middle frontal lobe. (B) ALFF differences between ALS and control groups with GM correction. There were significant decreased ALFF in visual cortex, fusiform gyrus and right postcentral gyrus, as well as increased ALFF in left middle frontal lobe and right inferior frontal gyrus. There were some slight changes after GM correction. For instance, after GM correction, the increased intrinsic brain activities in right inferior frontal gyrus reached significant level (marked by red arrow). Green indicates that ALS patients had decreased ALFF compared with the controls and the red indicates the opposite. The statistical threshold was set at voxel-wise t>3.32 (P<0.001) and cluster level of p<0.05 corrected by FWE.

By using voxel-based analysis without taking regional GM volume as covariates, compared to the controls, the ALS patients showed significantly decreased ALFF in the inferior occipital lobe, fusiform gyri, and right postcentral gyrus. Significantly increased ALFF were found in the left middle frontal gyrus. The peak voxels within those significant different clusters were shown in **Figure 1A** **and** **Table 2** **.**

**Table 2:** Regions Showing Significantly changed ALFF value in ALS Patients.

	*Anatomic regions*	*Brodmann area*	*Coordinates of peak voxel* *(X, Y, Z)* a	*Maximum T score*	*Number of voxels*	*P-corrected with FWE*
WithoutGMcorrection	*ALS>control*					
	Frontal_Mid_L	46	−29 32 32	5.58	52	0.015
	*ALS<control*					
	Postcentral_R	3	49 −26 47	−4.85	45	0.03
	Fusifrom	37	27 −51 −11	−5	60	0.008
	Occipital_Inf	18/19	−32 −82 −11	−6.52	642	<0.001
WithGMcorrection	*ALS>control*					
	Frontal_Inf_R	11	40 33 −7	5.43	37	0.012
	Frontal_Mid_L	46	−29 32 32	6.43	84	<0.001
	*ALS<control*					
	Postcentral_R	3	49 −26 47	−4.96	36	0.013
	Fusifrom	37	27 −51 −11	−5.08	50	0.002
	Occipital_Inf	18/19	−32 −82 −11	−6.28	522	<0.001

a X, Y and Z are location of peak voxel in the Talairach–Tournoux coordinate;

The statistical threshold was set at voxel-wise t>3.32 (P<0.001) and cluster level of p<0.05 corrected by FWE (family-wise error).

In the VBM analysis, no GM volume difference reached the significant level. While taking regional GM volume as covariates, we found that the results of ALFF were approximately consistent with those without GM correction. However, we also noted that the significant level and cluster size were slightly changed after GM correction. Besides, after GM correction, the increased intrinsic brain activities in right inferior frontal gyrus reached significant level **(Details in** **Fig. 1B** **,** **Table 2**).

**Figure 2:**
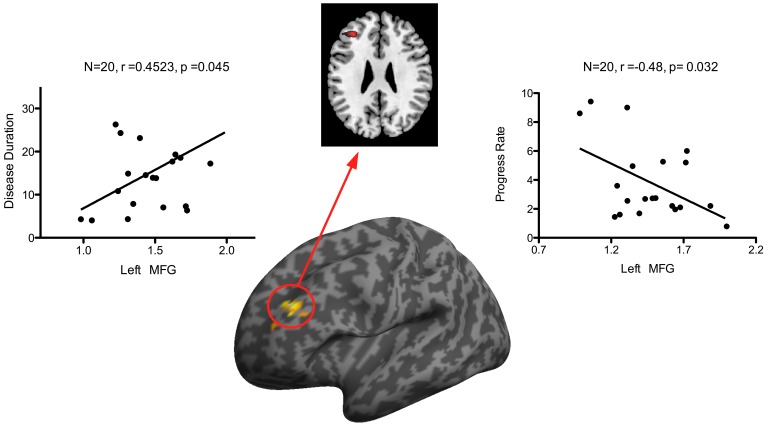
Correlations between ALFF value in left middle frontal gyrus and clinical measurements. Left middle frontal gyrus shows increased ALFF and scatter plots show correlations between ALFF of this area and disease progression rate and disease duration in the ALS group.

In ALS patients, a moderate positive correlation was identified between disease duration and the mean ALFF in the left MFG (r = −0.480, p = 0.038, **Figure 2**), while the rate of disease progression was negatively correlated with the mean ALFF in the left middle frontal gyrus (r = 0.452, p = 0.045, **Figure 2**). However, there was no significant correlation between the disease severity (ALSFRS scores) and the mean ALFF in the VOIs.

## Discussion

In the present study, we applied ALFF measurement to conduct a whole brain voxel-based analysis of cerebral function during the resting state, and our results revealed the abnormal neural activity outside the motor cortex in ALS patients which remained approximately consistent after controlling for the structural difference. Specifically, we found reduced ALFF in the inferior occipital lobe, bilateral fusiform gyri, and postcentral gyrus, while increased ALFF was found in left middle frontal gyrus, and in right inferior frontal gyrus after GM correction. Additionally, the ALFF value of the left middle frontal gyrus was negatively correlated with disease progression rate and positively correlated with disease duration. Our results suggest that the ALFF measurement of intrinsic brain activity could be useful to characterize the physiology of ALS.

Abnormal brain activities in the sensorymotor cortex have been consistently observed by position emission tomography (PET) studies [52], [53] or fMRI studies [13], [14], [16], [17]. Reduced grey matter volume identified in this area [54], [55] provided structural basis for the above findings. Our finding that ALS patients showed decreased ALFF in the postcentral gyrus was in line with these previous studies. Given that ALS is a degeneration disease predominantly affecting motor system, this alteration seems to reflect dysfunction in motor system in ALS patients. However, on the other hand, previous clinical observation studies [5], [56]–[58], electrophysiological studies[4], [59]–[62], and neuropathological studies [4], [63] have provided pathologic evidence for abnormalities in the sensory system. Since the peak voxel is located in postcentral gyrus––the main sensory receptive area for the sense of touch, it is possible that this alteration may reflect the dysfunction in sensory system.

Moreover, more widely decreased ALFF was found in the visual processing areas: the inferior occipital lobe and fusiform gyri. This finding suggests a disturbance of visual process, which is in accordance with previous electrophysiological studies and task-related fMRI studies. A reduced and delayed response to visual stimuli was observed in sensory cortical processing areas in ALS patients by electrophysiological studies [64], [65], and progressive reduced activation of extrastriate areas over the course of ALS was identified by a previous longitudinal study [66]. Combining fMRI with structural MRI, researchers demonstrated the existence of a decreased response in secondary visual areas in ALS during visual stimulation, and significantly less functional white fiber tracts projecting to visual areas [67]. Taken together, our findings strongly suggest that abnormalities in visual processing areas can be caused by the demyelination of nerve fibers in the optic tract [67].

On the whole, the decreased ALFF in the inferior occipital lobe, bilateral fusiform gyri and the right postcentral gyrus detected in the current study may suggest functional deficits in the sensory system of ALS. However, the decreased activities in the sensory system could also be a result of attenuated afferent peripheral inflow caused by progressive immobility in ALS [67]. It is unclear whether the progressive functional deficit in secondary/higher order sensory processing areas in ALS is a specific event in the expression of this disease process, or just an unspecific reaction to the reduction of external information flow. Future longitudinal studies on ALS are needed to clarify this question.

Apart from the reduced intrinsic activities, increased activities have been observed in the left middle frontal gyrus in our ALS patients. Besides, after GM correction, we noticed the increased intrinsic brain activities in right inferior fontal gyrus reached significance. Similarly, in a PET study [68], poor cognitive performance was associated with decreased binding in left middle frontal gyrus and right inferior frontal gyrus, indicating those areas playing an important role in the cognitive function. These increased activities in these areas are likely to reflect the compensatory process for dysfunction of frontoparietal network, which has been suggested to be related to cognitive function and be disrupted in ALS patients by a recent fMRI study [38]. In the early stages of ALS, the compensatory mechanism may temporarily “cover” the functional deficits. The patients in the present study were at a relatively early stage of the disease; this might explain why we didn’t found wide spread decreased brain activities in other frontal areas or motor areas. However, the compensatory mechanism is limited and might be exhausted with the increasing burden of disease pathology [23]. In more advanced patients, we anticipate that ALFF alterations will be more spread out in motor and frontal areas. This needs future longitudinal study to elucidate.

The negative correlation between the rate of disease progression and the ALFF value in the left middle frontal gyrus also gives support to the hypothesis that the increased brain activities in the left middle frontal gyrus act in a compensatory role. More wide-spread frontal lobe dysfunction could hinder the compensatory the process, which is supported by the fact that co-occurrence of frontotemporal dementia and even executive dysfunction are unfavorable prognostic factors in ALS [10]. This indicates that the ALFF value in the left middle frontal gyrus may be a quantitative marker in predicting the prognosis of ALS in the early stages of the disease.

No relation was found between the disease severity, measured by the ALSFRS, and the ALFF value in the abnormal brain areas. The reasonable explanations are listed as follows. The lack of objectivity was reported in clinical assessment using ALSFRS which can be masked by lower motor neuron signs [69]. Furthermore, we chose the ALSFRS instead of the revised vision––ALSFRS-R [70], which incorporates additional assessments of dyspnea, orthopnea, and the need for ventilator support. An objective upper motor neuron measure would contribute to the association between disease severity and ALFF measurements.

Although previous VBM studies showed grey matter volume decreased widely, especially in the motor cortex [54], [55], [71], [72] and frontal and temporal regions [54], [73], [74], we didn’t found significant grey matter atrophy in our sample. This may be due to a relatively early stage of our patients. After controlling for the regional grey matter volume, we found that the results of ALFF were slightly changed, but the patterns were approximately consistent with those without GM correction. Clarifying the interaction between structural difference and BOLD signals has been attempted by several recent fMRI studies [30], [47], [75]. Most of them found that the functional results retained mainly consistent but with changed significance after GM correction. Our results are in accordance with these previous studies and imply ALFF are reliable to reflect the functional abnormalities in intrinsic brain activities in ALS patients.

There are several limitations in this study. First, the sample size was relatively small, which limits the generalization of the results. A larger group of ALS patients needs to be recruited for long-term longitudinal observation. Second, lack of objective assessment of sensory function and more specific neuropsychological assessment of cognitive function in ALS patients hampers our interpretations of the results. Further investigations should be focused on the relationship between sensory deficits and abnormal spontaneous activity in the sensory cortex.

In conclusion, our observations confirmed the abnormal spontaneous brain activity in the sensorimotor cortex, visual cortex and frontal lobe; supporting the concept that ALS is a multisystem disorder. These observations would also extend the ALS phenotype to include sensory neuropathy. The ALFF value in the left middle frontal gyrus may be a quantitative marker in predicting the ALS prognosis.
